# ‘What can I do that will most help researchers?’ A different approach to training the public at the start of their involvement in research

**DOI:** 10.1186/s40900-019-0144-4

**Published:** 2019-02-20

**Authors:** Kristina Staley, Emma Cockcroft, Andrea Shelly, Kristin Liabo

**Affiliations:** University of Exeter Medical School South Cloisters, St Luke’s Campus, Exeter, EX1 2LU UK

**Keywords:** Public involvement, Training, Patient and public involvement, Co-production

## Abstract

For patients and the public to work collaboratively with researchers, they need support and opportunities to engage in learning that builds on their skills and grows their confidence. In this article, we argue for a different approach to this learning, which starts with the expertise patients/ the public arrive with, and helps them identify and develop the soft skills required to influence researchers effectively. Much of the current training for patients and the public focuses on addressing the gaps in their knowledge and awareness about how research works and how public involvement adds value. Our training complements this by exploring the concept of ‘experiential knowledge’ in more depth. Patients and the public possess experiential knowledge (knowledge gained through lived experience) that researchers may not have. In the training we explore the nature of this expertise and other skills that patients/ the public bring, as well as how to identify who has the most relevant experiential knowledge in any given situation, and how best to share experiential knowledge to benefit researchers and maximise the impact of involvement. We co-produced this training with a patient member of the project team, and through feedback from patients and carers in an initial pilot. Our approach adds another dimension to preparing people for involvement and in particular for taking part in conversations with researchers that support mutual learning. We suggest this approach should be supported by separate, mirror training for researchers, that also develops their soft skills in preparation for learning from involvement.

**Electronic supplementary material:**

The online version of this article (10.1186/s40900-019-0144-4) contains supplementary material, which is available to authorized users.

## Plain English summary

In this article, we propose a different approach to supporting patients and the public when they first become involved in research. We suggest that when people are new to involvement they need to be made aware of what they already know that is useful to researchers, and the soft skills required to be effective. Providing training to patients and the public is not a new idea. However, much of the current training explains how research works and how public involvement makes a difference. In contrast, our training helps people understand how best to share their knowledge in a way that benefits researchers and maximises their impact. We co-produced this training with a patient member of the project team, and through feedback from patients and carers. Our experience suggests that this training helps to build people’s confidence and to better understand how to draw on their experience and use it constructively. It helps to prepare patients and the public to take part in conversations with researchers that support two-way learning. In this article we describe how we developed this training. We conclude that this approach should be supported by separate, mirror training for researchers that also develops their soft skills in preparation for learning from involvement.

## Background

Training is increasingly considered an essential part of ‘good’ patient and public involvement^1^ in research. Recently published national standards on involvement in the UK emphasise providing support and learning to build people’s confidence and skills for involvement [1]. This is as important for researchers as it is for patients/ the public, as all stakeholders benefit from a greater understanding of how involvement works and how to work effectively together. However, in this article we particularly focus on the development of a different approach to training for patients and the public, as this was the first piece of work we carried out. It is our intention to develop mirror learning opportunities for researchers to address the issues we raise.

Current training of patients and members of the public often focuses on addressing the gaps in their knowledge and awareness about how research works and how public involvement adds value [2–5]. It describes good practice as well as the practical issues around being involved, and may also equip patients/ the public with the skills and knowledge to be involved in a particular task [6]. This training is very valuable and helps patients/ the public to understand the context in which they are working, as well as the purpose of their activity [4]. Through our collective work in recent years, we have identified additional learning needs amongst patients and the public, particularly at the early stages of their involvement. In this article we discuss how we identified these needs and then developed a training programme to respond to them. We suggest this offers an additional step in the provision of support and learning opportunities for public involvement in research.

### Why did we develop this training?

One rationale for this project came in 2017, when KS consulted a range of people with experience of being involved in research, to inform the revision of the Public Information Pack (PIP) produced by INVOLVE [7]. (Established 1996, INVOLVE is a government funded organisation in the UK, that supports active public involvement in NHS (national health service), public health and social care research.) The PIP information pack provides an introduction to involvement to people who are new, and part of the consultation asked experienced people what information they would have found useful at the start. Many said they would have benefited from a greater understanding of how research links to health service development and improved care, as well as a better appreciation of how their existing knowledge and experience is relevant and useful. They explained that they didn’t necessarily want to hear about the technical aspects of research at this early stage. This technical information can be easier to absorb on an ongoing basis, through different involvement projects (e.g. while working on a clinical trial, it can be easier to learn how a trial works within a real-life context). They found it confusing to hear mixed messages in their initial training. On the one hand they were told ‘*You don’t need to know anything about research to get involved*’, at the same time as hearing ‘*Here’s all this information about research that you do need to learn’.* Patients and carers involved in a study in Denmark, similarly commented that their initial training focused ‘*too much on research theories and approaches than roles and responsibilities*’ [8].

EC and KL are members of the involvement team of an applied health research programme [9] and support the involvement group of this programme, called PenPIG (Peninsula Public Involvement Group) [10]. PenPIG is a group of patients and carers who collaborate with researchers on developing research funding applications, and are members of research teams on funded studies. EC and KL had experienced situations in which members of PenPIG had put themselves forward for involvement opportunities, when these individuals did not have personal knowledge and experience of the study topic. This had sometimes led to conversations where patients and researchers had disagreed about what kind of patient experiences were needed to design a study. KL and EC therefore wanted to develop training that would help PenPIG members appreciate the need for different kinds of experience in different research contexts.

At the start of this project, AS was a relatively new member of PenPIG and felt uncertain about which parts of her story to share with researchers. She had many years’ experience of different health issues for herself and her family members, all of which could be potentially relevant, and she wanted guidance to help her identify which aspects of her story would be most useful to share. In the absence of this guidance, she thought that involved patients/ members of the public could fall into the pattern of telling the same ‘life story’ in every involvement situation, because ‘*they didn’t know, what the researchers wanted to know*’. KL and EC had observed interactions between researchers and PenPIG members where this had been the case.

Our combined experience therefore suggested to us that training for people who are new to involvement could usefully explore the concept of experiential knowledge in much more depth, to explain what it is, to identify who has relevant experiential knowledge in any given situation, and to understand how best to share this knowledge to the benefit of researchers and to maximise impact. (Experiential knowledge is knowledge gained through life i.e. wisdom, rather than knowledge gained through formal training or education). In this paper, we describe how we developed this new approach and the feedback from people who have taken part.

### Developing the training

The four authors of this paper formed a team to develop and deliver the new training programme. We met on several occasions over the course of a year to develop an initial programme that was tested with experienced patients/ members of the public, revised and then delivered to people who were new to research. The content and delivery of the training was thus co-produced, with the input of a wide range of patients/ members of the public, consistent with INVOLVE’s guidance [11]. EC drew on her experience as an Associate Fellow of the Higher Education Academy to help structure the training.

Initially, we discussed and agreed the learning outcomes. We hoped that by the end of the training, participants would have a better understanding of:

- Patient and public involvement and its importance to research
- How their contributions make a difference
- How best to draw on their experience to help researchers

In common with other training programmes [12], we wanted to start ‘where people are at’, to help them understand the value of what they already know, and to understand how to share their wisdom and experience in a way that researchers can hear. We wanted to focus on the importance and value of patients and the public sharing their existing experiential knowledge, rather than on filling any gaps in their technical knowledge about research. We therefore reflected on ‘what happens in the room’ when researchers and patients/ the public work together and considered what soft skills might be required to help patients/ the public to be effective. Soft skills are interpersonal skills which help people to work well with others and include attitude, communication, reflection, decision-making, positivity, time management, motivation, problem-solving, critical thinking, and resolving conflict [13]. We also considered the unique contributions that patients/ the public make through sharing the knowledge that only they possess from their lived experience [14, 15].

From our perspective, the impact of involvement often occurs through a process of mutual learning (people learning from each other through conversation and joint reflection). Patients/ the public have experiential knowledge (wisdom) gained from their lived experience that researchers will not have (unless they have the had the same lived experience). For researchers, learning often happens *in the moment* when involved patients/ the public share their experiential knowledge to fill gaps in the researchers’ understanding, and/ or challenge any assumptions they have made [16, 17]. This impact, a moment of insight, is often experienced by researchers as a ‘lightbulb moment’ [16, 18]. The precise detail of what any researcher learns depends on what they as an individual ‘didn’t know’ before involvement, as well as the knowledge, opinions, and values that patients/ members of the public share with them. Researchers often don’t know what they don’t know [19]. For this reason, it’s impossible to predict which aspects of the patient’s/ member of the public’s experiential knowledge (which part of their story) is going to be most useful to share ahead of time. For the involved patient/ member of the public, the task of involvement therefore requires finding out and identifying what the researcher doesn’t know or has assumed. To influence researchers, and act as critical friends, they then need to share their relevant knowledge/ experience in a way that is perceived as constructive and supportive. (A critical friend is someone who is encouraging and positive, but who also provides honest and often candid feedback that may be uncomfortable or difficult to hear). Learning often goes both ways [20] as patients/the public also learn more about research through involvement. However, for the purposes of this training for patients/ members of the public, we focused on what the researchers can learn from other’s experiential knowledge.

**Fig. 1:**
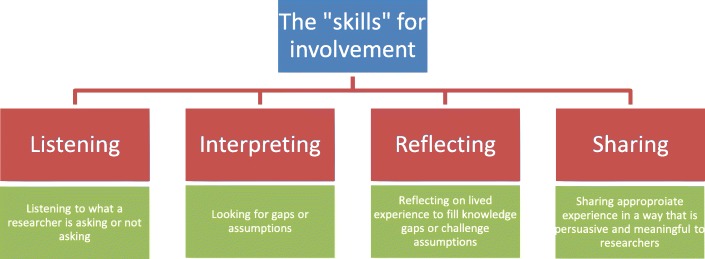
Some of the skills required by the public to be effective when involved in research

We concluded that the soft skills required by patients/ the public when working with researchers include (a) listening, (b) interpreting, (c) reflecting and (d) sharing, as shown in Fig. 1. From the perspective of the involved patient, to be effective they first need to listen to what the researcher is saying, to identify what questions are being asked, or what issues are not being discussed. They then have to interpret what is being said, to look for gaps in the researchers’ understanding and/or false assumptions. Reflecting on their experience enables them to identify the key information that researchers are missing and sharing that particular aspect of their experience (that particular part of their story) that helps to correct assumptions and provide learning. Thus ‘telling your story’ becomes a means to communicate and influence, rather than being an end in itself.

In our experience, this knowledge exchange often happens naturally through conversation, and many people have the innate skills to be able to do this. We believe that others may benefit from greater awareness of the steps involved, and from further support to enhance this set of skills. We therefore included an exploratory discussion of these skills for involvement in the final part of our training programme.

We developed the initial part of the training programme to provide the building blocks to support this final session on the soft skills of involvement. We developed training materials with the aim of making the session interactive, to require people use some of the skills we were highlighting and to minimise the number and length of presentations. At the end of the session, we supplied participants with the new INVOLVE PIP documents and signposted them to national and local contacts where they could obtain further information and support.

### Testing and delivering the training

We first tested the training programme with a group of 12 patients/ members of the public with 1–10 years’ experience of involvement in research and two involvement experts, asking them to act as critical friends. We ran the Programme as planned and at the end of each session invited participants to first ask any questions or comment on the content, and subsequently to reflect on whether they thought the session would be useful to people who were new to involvement and/or could be improved in anyway. As well as participating in a group discussion, the participants were asked to provide written anonymous feedback on each session. The four authors co-delivered the training and made notes based on observations.

This test provided very useful learning on how the training could be improved. Overall the feedback from participants was very positive, for example one person commented*, “The whole session/day was very good. Clear and concise and very well paced. Informative, stimulating, enjoyable and productive.”* We were also given a clear steer on changes that would make some of the content more accessible and relevant to people who were new. While much of the content was familiar to experienced people, many commented that they had learnt something new from the discussion around whose experience is most relevant for specific projects. This suggests this is a topic that would benefit from greater awareness and discussion.

We revised the training programme in light of the responses to this initial test, and reran the training, inviting people who were new to involvement. Six people attended, five of whom had had just started to get involved in research. All six participants reported having improved their understanding of public involvement in research, of why it’s important and how it makes a difference as well as how best to make a contribution. They all rated the delivery of the training very highly, for example commenting *“I felt I had a low understanding prior to this course, but it has now given me the confidence to know “I can do this”. Knowing my knowledge is beneficial to researchers and how much of an impact this can have.”* Five out of six said they would plan to get more involved in research after the training.

### Final programme content

Based on this development work, we have agreed a final programme (see Additional file 1). The first part of the programme (Session 1) consisted of a presentation where we introduced key concepts and terms and described experiential knowledge in terms of who has it and how it is of value to researchers. We also described a detailed example of a project where patients had contributed their experiential knowledge throughout and the impacts this had had on the researchers and the research. Session 2 was a matching exercise where participants matched summary statements of the ways in which sharing experiential knowledge helped researchers, with more detailed statements describing the knowledge that the public shared and the impact on researchers’ thinking and plans. This provided an an insight into the breadth of ways that involvement in research helps researchers. Session 3 was another presentation which discussed the issues around who are the right people to get involved in different kinds of research project, the different skills and experience the public can contribute, and the issues people might want to consider before agreeing to get involved. Session 4 was a practical exercise that helped participants to explore which patients/carers/members of the public and professionals should be involved in a research project, by discussing a real example. The final session took the form of a listening and reflective exercise where participants discussed the best ways to share their experiential knowledge and the challenges in doing this. They identified the skills required and reflected on any further learning or training they might need to enhance or develop these skills. At the end of the Programme, we summed up the key learning points from the session and signposted participants to local and national resources on involvement in research.

## Conclusions

Our experience of developing and delivering this training has confirmed that there is potentially a gap in current training for patients/ the public at the start of their involvement in research. People who have been involved for many years commented that although over time they have learnt what they can usefully contribute and how to have a greater influence, they would have benefited from these insights right from the beginning. Their experience of previous training was that it helped them understand generally why involvement is important and how involvement is carried out in practice, but still left them with uncertainty and concerns about what precisely would happen when they worked with researchers, and in particular *how* involvement works. Our approach aimed to make it more explicit how researchers learn from patients’ and the public experiential knowledge and to prepare people for sharing aspects of their story as a way of persuading and influencing. We believe this adds another dimension to preparing people for conversations with researchers that support mutual learning. The feedback from the participants confirmed that they felt more confident about involvement, were clearer about what to expect and had gained a better understanding of how to draw on their experience and use it constructively.

In the discussion sessions during the training, some participants asked whether it is possible to train people in the soft skills required for effective involvement, i.e. listening, interpreting, reflecting and communicating skills. We believe, as with all skills, that everyone will be on a spectrum in terms of their ability, and there may some benefit from training to develop or enhance these particular skills. Our training programme, lasting only 3–4 h, sought to raise awareness of the need for these skills and to illustrate ‘helpful’ and ‘unhelpful’ practice. Some patients/ members of the public might require additional training and support to address their specific skills gap. As we explained during our sessions, learning how to be effective in public involvement is an ongoing process, and we all continue to learn through our experience over a number of years. Learning ‘how’ to do it well, may not always require formal training, but might equally be supported through mentoring, coaching as well as ‘learning on the job’. What we hoped to achieve through our training was to bring more awareness to the learning conversations that underpin involvement, so that people can be mindful of this in their interactions with researchers and as part of their personal development over time.

It was notable that participants identified a number of ways in which researchers could support the development of the patients/ public’s skills and provide feedback as to what kinds of contributions are most useful. Not everybody will always be precise and clear in sharing their experience, so researchers need to be able to work with a range of different people and know how to get the best from all of them. We are developing a mirror training programme for researchers, which will focus on their role and responsibilities in enabling a meaningful, mutual learning experience. We are also aware that involvement leads/ facilitators can play an important role here, with implications for the knowledge and skills they require to be effective.

We considered whether there was a case for training patients/ the public and researchers together at this stage. However, we believe that the starting points for patients/ members of the public and researchers are quite different. There are different messages for each of them about how involvement relates to what they already know, and the skills they might need to develop. We therefore concluded that there may be benefits to the two groups working separately to better prepare themselves *before* they start working together, so that when they do start collaborating, they are much more effective. At later stages, joint training may be more appropriate, for example, EC is working with patients/ members of the public to develop joint training for researchers and patients/ public to support effective team working.

It was of note, that many of the experienced patients and carers reported learning something new from a discussion of whose experiential knowledge is most relevant in different contexts. We recognise this can be a thorny issue, with researchers and involvement leads feeling uncomfortable and uncertain about when to say ‘no’ to people who put themselves forward. There may be value in increasing awareness and dialogue on this topic. Patients and members of the public may have an important role to play in helping to answer this question for researchers. Given our experience of the problems that can occur if people who lack the relevant experience are involved [20], we believe that all parties will benefit from further consideration of who is most appropriate to involve and why. Addressing this issue could enhance the impact of involvement and ensure a better quality learning experience for all involved.

## Additional file

Additional file 1: Outline Programme for 'An Introduction to Patient and Public Involvement in Research'. (PDF 31 kb)

## Abbreviations

NHS: National Health Service
PenPIG: Peninsula Public Involvement Group

## Footnotes

1 We use the term ‘patient and public’ to refer to people with relevant experiential knowledge to contribute to research. Depending on the context, this could be patients, carers, potential and current users of services and/ or community members. We use the term involvement to refer to the activities whereby these people act as collaborators in research influencing its design, delivery and dissemination.
