# Supplementary material for: ‘What can I do that will most help researchers?’ A different approach to training the public at the start of their involvement in research
Source: Res Involv Engagem. 2019 Feb 20;5:10. doi: 10.1186/s40900-019-0144-4 (PMC6383232; doi:10.1186/s40900-019-0144-4)
Supplement: Supplementary file 1 — Outline Programme for 'An Introduction to Patient and Public Involvement in Research'. (PDF 31 kb) [file 40900_2019_144_MOESM1_ESM.pdf]

NIHR CLAHRC
South West Peninsula

NHS
National Institute for Health Research

TwoCan ASSOCIATES

An Introduction to Patient and Public Involvement in Research

10:30-15:00 Exeter Medical School Room G25

Programme

**10:30**	**Arrival: Coffee and Tea**
**11:00**	**Welcome and Introductions**
**11:10**	**Session 1: What is patient and public involvement in research and why is it important?**
**11:40**	**Session 2: What difference does involvement make?**
**12:00**	**BREAK**
**12:10**	**Session 3: Finding the right opportunity for you**
**12:30**	**Session 4: Who has the right knowledge and experience to be involved?**
**13:05**	**LUNCH**
**13:45**	**Session 5: The skills required for involvement**
**14:45**	**Final feedback**
**15:00**	**END**
